# Motor imagery training for children with developmental coordination disorder – study protocol for a randomized controlled trial

**DOI:** 10.1186/s12883-016-0530-6

**Published:** 2016-01-12

**Authors:** Imke L. J. Adams, Bert Steenbergen, Jessica M. Lust, Bouwien C. M. Smits-Engelsman

**Affiliations:** Behavioural Science Institute, Radboud University Nijmegen, PO Box 9104, 6500 HE Nijmegen, The Netherlands; School of Psychology, Australian Catholic University, Melbourne, 3065 VIC Australia; Department of Health and Rehabilitation Sciences Faculty of Health Sciences, University of Cape Town, Old Main Building Grote Schuur Hospital, Cape Town, South Africa

**Keywords:** Developmental coordination disorder, DCD, Internal modeling deficit, Motor imagery, Motor imagery training, Cognitive orientation to daily occupational performance, CO-OP

## Abstract

**Background:**

Previous studies have shown that the predictive control of movements is impaired in children with Developmental Coordination Disorder (DCD), most likely due to a deficit in the internal modeling of movements. Motor imagery paradigms have been used to test this internal modeling deficit. The aim of the present study is to examine whether a training focused on the mental imagery of motor skills, can help to improve the motor abilities of children with DCD.

**Methods/Design:**

A pre-post design will be used to examine the motor performance, motor imagery and motor planning abilities before and after a training of 9 weeks. Two groups will be included in this study (1) one receiving motor imagery (MI) training focused on the forward modeling of purposive actions, (2) one receiving Cognitive Orientation to daily Occupational Performance (CO-OP) training focused on identifying effective cognitive strategies that will increase motor competence. MI training will be given with the use of instruction videos of the motor skill that will be trained. Both groups will participate in 9 individual sessions of 45 min (once a week) with a paediatric physical or occupational therapist, added with homework sessions. Inclusion criteria are: (1) aged 7–12 years, (2) meeting the DSM-V criteria for DCD (motor performance substantially low (score on the m-ABC ≤ 16th percentile) and motor problems that interfere with daily life (DCDQ, and request for help at a paediatric physical or occupational therapist)). Exclusion criteria are IQ < 70 and other medical conditions causing the motor impairment.

**Discussion:**

The results of this study will help to make treatment protocols for children with DCD more evidence-based. This study will increase our knowledge about the efficacy of both the MI training and CO-OP training, and both children with DCD and therapists will benefit from this knowledge.

**Trial registration:**

www.trialregister.nl/NTR5471.

## Background

Children with Developmental Coordination Disorder (DCD) show motor performance that is substantially below expected levels, given the child’s chronologic age and previous opportunities for skill learning [1]. The prevalence estimate for DCD is 5–6 % [2], and a prerequisite for a diagnosis DCD is that these problems with motor skills are significant enough to interfere with both social and academic functioning. The etiology of DCD has been examined in several studies which reveal a number of viable hypotheses including reduced processing speed, problems in executive functioning, poor cross-model integration and poor perceptual-motor coupling (for review see [3]). In two recent systematic reviews [3, 4] this collective evidence was shown to reveal an underlying deficit in motor control and learning, linked to the predictive control of movements. This deficit has also been described as the ‘internal modeling deficit’ (IMD) [5] and is thought to compromise the motor learning capabilities of children with DCD. Internal models provide stability to the motor system by predicting the outcome of movements before slow, sensori-motor feedback becomes available [6], providing a means of rapid online correction [7, 8] and anticipatory control. We recently showed that children with DCD indeed experience problems with tasks that are thought to rely on an internal model of a movement - motor imagery, action planning and rapid online control of movements [4]. The impaired performance on these experimental tasks in children with DCD might be caused by an inaccurate or incomplete internal model of movements. It is crucial that children with DCD learn to make a comparison between the predicted and actual sensory feedback, and thereby learn to fine-tune their internal model of several movements. A motor imagery (MI) training, which is focused on the comparison between the predicted consequences of a movement (by using imagery) and the actual consequences of a movement, might help children with DCD to improve the predictive control of movements.

Currently, three main professions provide treatment for children with DCD: occupational therapy, physical therapy and special education [9]. Occupational therapists analyze capacities and performance and develop intervention and therapy solutions for problems related to performance and participation in close co-operation with the child and parents. Physical therapists help children to develop and optimize their mobility and movement-related functions. Educational approaches are not discussed in this study protocol because these approaches are mainly focused on improving school activities and less focused on improving motor skills. Occupational therapists and physical therapists both use strategic task-oriented approaches like Cognitive Orientation to daily Occupation Performance (CO-OP) [10] and also specific task-oriented interventions like neuromotor task training (NTT) [11]. Both approaches, CO-OP and NTT, are often used in the treatment of children with DCD [12]. CO-OP focuses on performance of the activities that a child needs or wants to master. CO-OP involves improvement of knowledge of the task, cognitive strategy use, learning and teaching principles, self-instruction, adaptation of environment and involves the Goal-Plan-Do-Check framework [10, 13]. Several studies have shown that the CO-OP intervention is effective to improve motor performance in children with DCD [10, 14–16]. Recently, Thornton and colleagues [17] showed that CO-OP intervention can also be effective in a group environment. In NTT, skills are examined through task analysis [18]. A task is broken down into its component parts if necessary and this will enable to focus on the main problems in the task [11]. First, the tasks and activities related to participation, which are of greatest concern to the child, and his family, need to be identified and tasks or activities for the training need to be selected. By using motor teaching strategies, therapists guide children through the different phases of motor skill learning by gradually increasing task demands. Task and environmental constraints that impede successful task performance are identified and manipulated in intervention sessions to provide the opportunity to practice and improve the deficient motor skills [19]. NTT was shown to yield positive (task-specific) changes on measures of gross- and fine-motor skill [11, 20, 21].

To make children with DCD more aware of how they can predict the consequences of executed movements, and the comparison that can be made between predicted and actual sensory feedback, MI training can be used. MI training was first and only used in children with a lower score for motor skills (Movement assessment battery for children (m-ABC) percentile score < 50^th^) by Wilson and colleagues [22]. This study showed that after 5 h of individual training, the MI training group significantly improved their motor skills as measured by the movement ABC, while the wait list control group showed no significant improvements. Motor imagery involves the imagination of moving specific body parts without the actual movement of those parts. During motor imagery, the participant is asked to imagine making a certain movement, which is expected to facilitate the participant in predicting the consequences of actions in absence of the overt movement. In combination with continued actual practice, participants use the knowledge of the relation between vision and kinaesthesis to make accurate predictions of the consequences of self-produced movements, which will reduce the errors in feedforward planning [9]. MI training can help to build motor representations that are needed to improve predictive control. Notably, in athletes, a structured program of motor imagery can lead to an improvement of performance [23] and also in a rehabilitation context it has been proven that MI training can enhance motor recovery after stroke [24]. In the systematic review of Schuster et al. [25] it was indicated that MI training is evaluated in more than 100 studies with adults. However, only a restricted number of studies have used MI training in children [25]. An important difference in MI training used in athletes and post-stroke patients compared to its use in children is that the former group of individuals are capable or have been capable before to perform a selected movement. In children, MI training is used to learn motor abilities that they do not yet master. The rationale for using MI training to promote the (re)learning of motor function arises from the functional correlates that MI shares with the execution of physical movements. It is now recognized that the duration of mentally simulated actions usually correlates well with the duration of real movements, indicating that the simulation of movements evokes similar autonomic responses and that the imagination of an action or its physical execution engage largely similar neural networks [26–28]. MI training has already been described as an approach for children with DCD, but it is not recommended yet because there is a lack of solid empirical evidence [9]. In addition, in the study of Wilson et al. [22], only 61 % of the sample scored below the 15th percentile. The present study will be the first study that examines the effectiveness of a MI training protocol in children who meet the DSM-V diagnostic criteria for DCD. The objectives of this study are:

1. To study the effectiveness of MI training compared to the in the EACD guideline recommended CO-OP training for improving the motor abilities of children with DCD;
2. To examine the relation between MI ability and improvement in the motor abilities via MI training in children with DCD;
3. To assess patient and therapist satisfaction when applying MI training

## Methods/Design

### Design

**Fig. 1:**
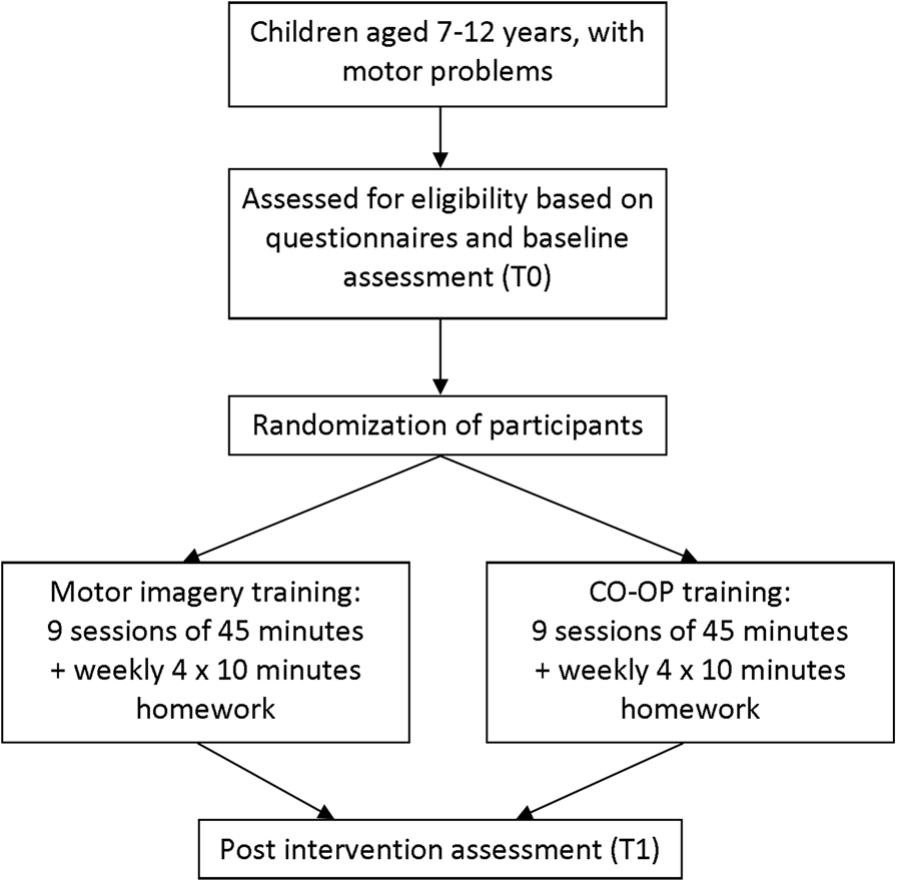
Flow of patients through the study

This study is a randomized controlled multicenter trial with two rehabilitation centers and 17 private practices for occupational and physical therapy across the Netherlands who are willing to co-operate. Therapists will participate in an instructive workshop about the MI or CO-OP training. MI training will be compared to CO-OP training, a recommended therapy for children with DCD. MI therapists will participate in an instructional course of 3 h, before the start of the study. CO-OP therapists have followed a two-day CO-OP training as designed by Polatajko and Mandich [29]. For the present study they will also participate in a short training of 1.5 h to update their knowledge of the CO-OP training and assure a standardized protocol. Children that meet the inclusion criteria for this study, will be randomly allocated to either the MI or CO-OP training group, see Fig. 1. Children in the MI and CO-OP group will receive the same amount therapist contact and training time as well as homework exercises.

### Patient population

All children between the ages of 7 and 12 years admitted at the rehabilitation centers and private practices for occupational and physical therapy for training of their motor abilities in the participating centers will be considered for inclusion. They will be offered participation in the study if they fulfill all of the following criteria (according to the DSM-V criteria for DCD [1]):

- Motor ability substantially below expected level given the chronological age of the child (criterion A). The Movement Assessment Battery for Children (m-ABC - 2^nd^ edition) will be used to assess the motor abilities [30]. A total percentile score ≤ 16^th^ or a component score ≤ 5^th^ is needed for inclusion.
- Motor impairment significantly interferes with daily life and/or academic achievement (criterion B). Only children that are referred to the centers for training of their motor abilities are included in this study. Referral for training of motor abilities is a strong indication that the motor impairment either causes problems in daily life, or with academic achievement. In addition, as recommended, the DCD Questionnaire (DCDQ) will be used to assess whether the motor impairment has an impact on the daily life or academic achievement of the child (Dutch translation DCD-Q [31]).
- Onset of symptoms is in the early developmental period (criterion C) as evidenced by their referral to a centre for training of their motor abilities between the ages of 7–12 years.
- No medical condition that could cause the motor impairment is known and IQ ≥ 70 (criterion D). This will be checked by using a health questionnaire that will be filled in by the parents/caregivers of the participating children. If parents do not report learning difficulties and the child attends a regular primary school, an IQ ≥ 70 is assumed. When attending special education, parents are asked to fill in the latest IQ score of their child.

In addition to the above mentioned criteria, attentional problems are assessed by using the attention deficit hyperactivity disorder (ADHD) questionnaire [32]. Parents/caregivers of participating children will be asked to fill in this questionnaire to be able to check in the off-line analysis if attentional problems are a confounder.

### Interventions

Both the MI and CO-OP intervention will be delivered for 9 weeks with 1 training session per week of 45 min. Additionally, participating children receive a homework booklet from their therapist and are required to practice 4 times a week for 10 min at home. Homework will be recorded by use of a diary. The number of 9 sessions was chosen because earlier studies on MI training in children with DCD [22] and CO-OP training [10, 15, 17] showed a training effect in the intervention group using 5 – 10 sessions. The 1 session per week was chosen as this was the most feasible schedule in both the rehabilitation centers and private practices, and has also been used in earlier studies on MI training [22]. For the CO-OP training it is not specified whether sessions should be once a week or more or less frequent [29, 33], but a recent study showing the effectiveness of a CO-OP intervention has also used sessions once a week [17]. Two self-chosen skills that are important for the child to learn will be selected for training during the 9 week intervention period [9]. The Motor Coordination Questionnaire will be filled in by both the parents and the children (guided by the therapist) to examine which motor skills are difficult for the child to perform (part A), and which motor skills are important for the child to master (part B). Using these two parts of the questionnaire, the therapist will help the child to choose two skills that will be targeted during the intervention period.

### Motor imagery training

**Fig. 2:**
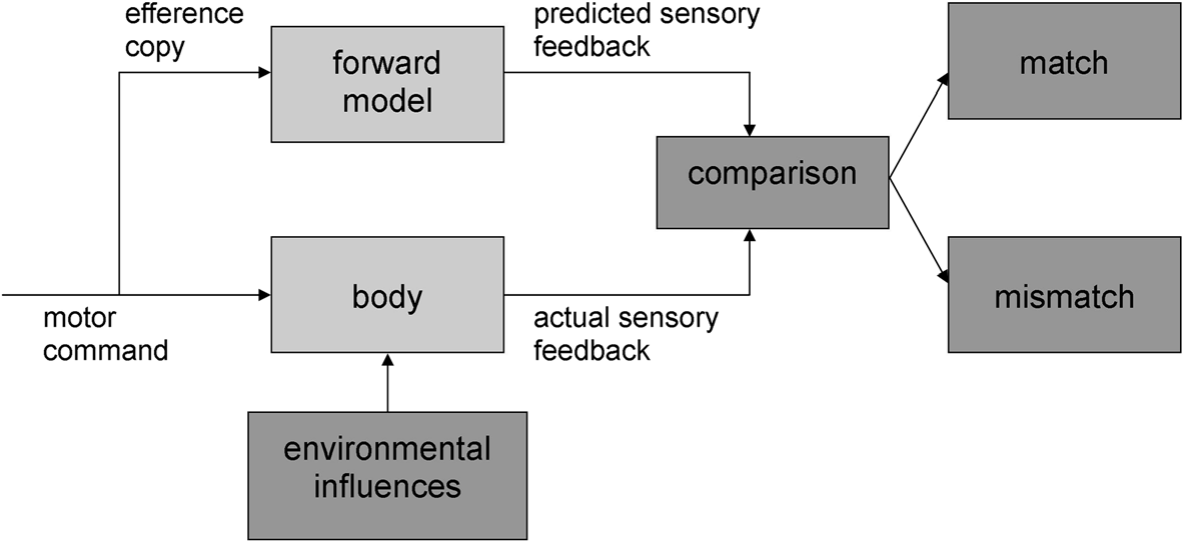
Forward model of motor control (adapted from [61])

In the review of Malouin, Jackson and Richards [34] three modes of MI delivery are discussed: (1) the motor imagery and physical practice are provided in separate sessions with MI training delivered either through audiotaped (or videotaped) scripts or guided by a therapist on a one to one basis, (2) motor imagery and physical practice are provided in the same session with series of physical repetitions alternating with the mental repetitions, (3) motor imagery alone. In a study of Courtine et al. [35], it was found that the timing (functional equivalence) of the motor task that is mentally rehearsed improved when motor imagery was alternated with physical repetitions within the same session. The process of forward internal modeling is depicted schematically in Fig. 2. Internal modeling comprises two aspects: an inverse modeling process that maps the necessary motor parameters (e.g. force, timing, trajectory) to achieve a desired goal state, and forward modeling that uses a predictive estimate of the sensory consequences of an action as means of error correction [6]. The output of the forward model provides a template against which real-time feedback can be compared under tight temporal constraints, and motor output signals can be corrected if needed [36]. When a mismatch occurs between predicted and actually sensory feedback an error signal is generated which provides an opportunity to correct movements online, but also helps to make the forward model for the next movement more accurate [35]. This suggests that afferent information during actual execution of a movement is helpful for consistent reproduction of the next imagined movement. Therefore, in the current study a combination of motor imagery and physical practice is used within the same session.

In addition, the PETTLEP model was used to design the current MI training. The PETLEPP model was developed by Holmes and Collins in 2001 [37]. The model is based on neuroscience research findings, particularly the discovery that the same neurophysiological processes underlie imagery and actual movement [38]. This ‘functional equivalence’ provides a possible explanation for the improvements of performance after imagery training [39]. The PETTLEP acronym relates to important components when implementing motor-based imagery interventions, namely: Physical, Environment, Task, Timing, Learning, Emotion and Perspective components. These seven components, as described by Holmes and Collins [37], are summarized here:

(I).  Physical: imagery is more effective when it includes all of the senses that would be engaged, and kinesthetic sensations that would be experienced, during actual performance.
(II).  Environment: It is important to imagine the performance in an environment that is as similar as possible to the actual performing environment, to be able to access the same motor representation. If a similar environment is not possible, photographs or videotapes can be used [40].
(III).  Task: The imagined task needs to be closely matched to the actual task. The participant should be encouraged to verbally report physiological and behavioral involvement, to emphasize a kinesthetic orientation toward the imagery [41].
(IV).  Timing: Equivalence in timing between the imagined and executed movement is important. To be able to access the same motor representation during motor imagery as during the execution of a movement, the temporal characteristics should be the same [37].
(V).  Learning: The imagery content needs to be adapted to the skill learning stage that the performer is currently in and moves from cognitive to autonomous. First the performer will have to think more about the technique, but in later stages of imagery the performer can focus more on the ‘feel’ of the movement [41].
(VI).  Emotion: To achieve optimal functional equivalence, the person should try to experience all of the emotions associated with the performance. This is in accordance with Lang [42] and Cuthbert, Vrana and Bradley [43] who suggest that the performer’s emotional responses must be included in imagery. The affective response during motor imagery is best shown through the autonomic system [44]. When faced with a physiological challenge, heart rate and respiration rate change already during motor preparation and subsequently in execution that reflect alterations in the energetic state of the performer [45].
(VII).  Perspective: From a functional equivalence perspective, imagery from a 1st person perspective (a representation of the self in action) is preferable because it is more closely related to the performer’s view when actual performing the movement.

In the current study, these PETLEPP elements are incorporated by using videos of two self-chosen skills that are important for the child to learn. The physical and environmental components are included by using these videos but also by coaching of the therapist to encourage including senses such as touch and hearing. The task and timing component are shown in the video and the participant should try to imagine the task that is displayed in the video and timing should be closely matched to the actual performance. The learning component is reflected in the video, in which motor skills are shown and subdivided into constituent sub movements or parts of the task. For instance in the video for writing, it is shown first how to sit at the table, secondly how to position the paper on the table, thirdly to ensure that the tip of the pen can be seen, fourthly to ensure that the pen moves smoothly over the paper and fifthly how to ensure that the paper does not move while writing. In addition, subsequent levels of the motor skill are shown in the videos. The therapist will encourage the child to also include emotional responses in the imagery, the emotional component, to make the imagery more vivid (for example, the therapist can ask the child how he feels during successful and unsuccessful performance). The perspective component is reflected because videos are first shown from a 3^rd^ person perspective, followed by a video from a 1^st^ person perspective, to evoke a vivid representation of the selected motor skill. The videos from a 3^rd^ person perspective are shown to allow the performer to ‘see’ which positions and movements are needed to perform the skill [46]. This also reflects to the learning component of the PETLEPP model, participants can first focus on the technique of the movement by watching the video from a 3^rd^ person perspective, followed by the video from a 1^st^ person perspective where they can focus more on the ‘feel’ of the movement as if they were doing it themselves.

The training protocol consists of several parts that will be run through every training session: (a) discuss homework of the past week and determine goal of current session (10 min), (b) watch videos of selected motor skill from 3^rd^ person perspective and 1^st^ person perspective, followed by mental rehearsal of this motor skill (10 min), (c) overt practice of the motor skill (10 min), (d) alternating mental rehearsal and overt practice of the motor skill, and compare and reflect on overt practice and mental rehearsal (10 min); (e) explaining homework for next week, advice for parents to motivate their child, goal of upcoming week (5 min). Videos of the following motor skills are provided by the researchers to the therapists: (a) Running and playing tag, (b) Throwing and catching a ball, (c) Hopping and playing hopscotch, (d) Jumping (amongst others rope skipping), (e) Bicycling, (f) Playing baseball, (g) Playing tennis, (h) Handwriting and (i) Eating with cutlery. On the videos the performance of the skill by a child aged 7–12 years is shown. If therapists want to train another motor skill with the child, they can record a short video themselves. Time practicing the two selected motor skills will be evenly distributed.

### CO-OP training

Like the MI training, the CO-OP training will focus on the acquisition of two self-chosen skills that are important for the child to learn. CO-OP is expected to improve the knowledge of the task through cognitive strategy use. CO-OP approach is based on cognitive behavior modification theories, in particular the verbal self-instruction strategy developed by Meichenbaum [47]. During a CO-OP intervention, a child learns this self-instruction strategy, which enables the child to identify why the performance was not successful, and to invent and execute plans to correct his/her performance (the goal-plan-do-check strategy) [13]. It is based on the belief that when a child guides himself through a problem-solving task by talking aloud, he/she learns to regulate behaviour by learning how to identify a goal, develop a plan and evaluate the success of that plan [48]. The training protocol consists of several parts that will be run through every training session: (a) discuss homework last week and determine goal of current session (10 min), (b) practice the selected motor skill using the Goal-Plan-Do-Check framework (30 min), (c) explaining homework for next week, tips for parents to motivate their child, goal of upcoming week (5 min). Time-on-task of both selected motor skills will be evenly distributed.

### Outcome measures

All tests will be performed by one assessor who is blind to group allocation at baseline (< 2 weeks before the intervention start) and post treatment (within < 2 weeks after last treatment). The assessor will be trained in the testing procedures before the start of this study.

### Primary outcome measure – m-ABC-2

The primary outcome measure is the score on the m-ABC-2 (Dutch translation [30]), reflecting the fine motor skills, gross motor skills and coordination abilities.

### Secondary outcome measures

#### Motor coordination questionnaire

Parents will be asked to fill in a questionnaire about 16 motor skills before and after the intervention, the Motor Coordination Questionnaire. Prior to the start of the intervention, they are asked how well their child is at performing these motor skills (part A), and how important it is for their child to perform well on these motor skills (part B). Children are also asked to fill out this questionnaire during the first training session. Parents and children can complement the questionnaire, with motor skills that are not present on the questionnaire, but important for their child to master. The answers help to choose the motor skills that will be trained during the intervention. After the intervention, parents and children will be asked to fill out this questionnaire again, however in addition to questions focusing on how well the child is able to perform the motor skills (part A), it is asked whether they think that the child has become better or worse in performing these motor skills (part C). All answers need to be filled out on a 5-point Likert scale. The score on the Motor Coordination Questionnaire will serve as an evaluation of the perceived improvement of motor skills of both participating children and their parents.

#### Video- analysis of the trained motor skills

Because motor learning is highly task-specific, general motor tests may not fully capture the change in trained skills that have few elements in common with the test items (for instance riding a bicycle). Therefore, the two self-selected motor skills will also be video recorded at the beginning of the first training session and at the end of the last training session. An independent assessor that is blind to group allocation and the time of recording will score the critical differences in execution of these skills. These outcome parameters will be more specific to the two trained motor skills than the m-ABC-2 score. In case the trained skill is handwriting, a standardized handwriting test (SOS-2 [49, 50]) will be used to evaluate the training effect.

#### Motor imagery performance

In earlier studies several tasks have been used that are thought to rely on an internal model of a movement – motor imagery, action planning and rapid online control of movements. Motor imagery performance will be tested with two tasks in this study, the hand rotation task [51] and the radial visual guided pointing task [52]. These two tasks are used because they are based on distinct concepts. In the hand rotation task an on-line, real-time representation of the body position is used, also called the ‘body schema’ [53, 54]. In contrast, in the radial visual guided pointing task an internal representation of the pointing movement is used to determine how long it takes to perform the task mentally, and this representation is mainly built by repeating practice and is less based on the body schema.

#### Action planning performance

In addition, a task to measure anticipatory action planning is assessed. Action planning can be defined as the ability to take the constraints of the action task and its goal into account when first taking hold of an object [55]. Tasks that examine action planning are also thought to rely on an internal model of a movement. We will use the sword task [56, 57], that has been previously used and validated in children. This task is specifically designed to measure action planning in children.

#### Rapid online control

The online control of movements requires that the motor system predicts the future location of the moving limb using a forward internal model [6, 58]. During target-directed reaching, the nervous system must implement rapid changes in trajectory in-flight, if the movement is perturbed in some way or in the event of a visually detected change in the environment. Experimentally, the operation of these internal feedback loops has been examined in children with DCD using double-step perturbation paradigms [7, 8]. In this study, we will also examine the detection and correction of online perturbations with the double step reaching paradigm.

#### Feasibility of MI training protocol

Therapists that have provided the MI or CO-OP will be asked to share their experiences and suggestions for improvement of the training after the intervention period. In addition, we will ask the children to fill in the Enjoyment Scale after the intervention period. The Enjoyment Scale is a 5 point scale with smiley faces (0 is no fun at all; 4 is super fun) that has been developed by Jelsma et al. [59]. Using the scale, we can examine whether children enjoyed the therapy, and if this is related to the overall performance score.

### Ethical approval

The MI training study has been approved by the Committee on Research Involving Human Subjects of the region Arnhem-Nijmegen in the Netherlands (protocol number 2013/463) and will be conducted in conformance with the ‘Declaration of Helsinki’. Written informed consent for participation of the parents of the children will be obtained. The trial is registered at the Dutch trial register, www.trialregister.nl (NTR5471).

### Sample size

The smallest detectable difference (SDD 95 %), regarded as clinically relevant of the m-ABC-2 as reported in the manual is two standard scores [30]. Using the SDD 95 % as the cut off, it will be established how many of the children improved their total standard score on the m-ABC-2 in the two groups. The number of participants is based on this SDD 95 %, with a statistical power of 80 % and an α = 5 % (two-tailed). Because the main question is whether the MI training group has a greater improvement of motor abilities than the CO-OP training group after the intervention, the sample size calculation is based on the difference between the MI and CO-OP training on the m-ABC-2 change score (before and after training). Power calculations yielded a required sample size of 58 participants, 29 participants in each group.

### Statistical analysis

Statistical methods to assess differences between groups will be ANOVA and ANCOVA analyses and Mann Whitney U tests. Between group comparisons will be made for primary and secondary outcome measures. Effect sizes (D) will be calculated to determine the practical significance of these differences. D-values greater than 0.5 indicate a moderate and values greater than 0.8 will indicate a large practical significance [60]. Potential confounders, such as age, gender and score on the ADHD questionnaire, will be included in an ANCOVA.

### Study organization

The study is organized and coordinated by the Radboud University in Nijmegen, the Netherlands. Collaborating institutions are 2 rehabilitation centers in the Netherlands and 17 private practices for occupational and physical therapy across the Netherlands.

## Discussion

MI training is already described as a possible treatment for children with DCD [9], but it is not recommended yet because there is only one study available [22]. The study of Wilson et al. [22] showed training effects comparable to conventional physical therapy. However, 39 % of the sample had a test score within the low normal range on the m-ABC. Therefore, conclusions about MI training for DCD should be interpreted with care. It is important to study whether MI training is also effective in a population with scores in the clinical range of the m-ABC-2 that meet the DSM-V diagnostic criteria for DCD.

Moreover, the multicenter character of the study will increase the generalizability of study results across different rehabilitation centers and private practices for occupational and physical therapy. The present study will also provide an opportunity to evaluate the feasibility of MI training both from the therapists’ and children’s point of view. As a result, the present study will also help to gather information needed to implement MI training on a larger scale. This study will increase our knowledge about the efficacy of both the MI training and CO-OP training, and therefore will help to make treatment protocols for children with DCD more evidence-based, from which both children with DCD and therapists will benefit.

## Abbreviations

ADHD: attention deficit hyperactivity disorder
ANCOVA: analysis of co-variance
ANOVA: analysis of variance
CO-OP: cognitive orientation to daily occupational performance
DCD: developmental coordination disorder
DCDQ: developmental coordination disorder questionnaire
DSM: diagnostic and statistical manual of mental disorders
EACD: European Academy for Childhood Disability
IMD: internal modeling deficit
m-ABC: movement assessment battery for children
MI: motor imagery
NTT: neuromotor task training
SDD: smallest detectable difference
SOS: systematische opsporing schrijfproblemen
